# Placental neutrophil reverse trans-migration and maternal serum neutrophil extracellular trap expression in HIV infection co-morbid pre-eclampsia in women of African ancestry

**DOI:** 10.1007/s00418-024-02298-6

**Published:** 2024-06-24

**Authors:** Merantha Moodley, Jagidesa Moodley, Thajasvarie Naicker

**Affiliations:** 1 Department of Obstetrics and Gynecology, School of Clinical Medicine, Women’s Health and HIV Research Group, College of Health Sciences, University of KwaZulu-Natal, Durban, 4041 South Africa; 2 Optics & Imaging Centre, Doris Duke Medical Research Institute, College of Health Sciences, University of KwaZulu-Natal, Durban, 4041 South Africa

**Keywords:** Human immunodeficiency virus, Neutrophil extracellular traps, Neutrophil reverse transmigration, Pre-eclampsia

## Abstract

Neutrophil extracellular traps (NETs) and placental neutrophil reverse transmigration (r-TM) are implicated in the pathogenesis of pre-eclampsia (PE). However, the role of the comorbidity of PE and human immunodeficiency virus (HIV) infection in placental neutrophil r-TM and serum NETs remains unknown. Human placental tissue (*n* = 160) and serum (*n* = 80) samples were obtained post-ethical approval and divided by pregnancy type and HIV status and across the study population. Immunohistochemistry and morphometry were performed to localize and quantify junctional adhesion molecule-C (JAM-C) expression as an inverse marker of neutrophil r-TM within placental villi. An enzyme-linked immunosorbent assay (ELISA) was performed to quantify the concentration of citrullinated histone H3 (cit-H3) as a marker of NETs. GraphPad Prism (version 8.0.2) was used to compare the results, and a *p* value of *p* < 0.05 was considered statistically significant. The localization of JAM-C was observed on the syncytiotrophoblasts (STBs) and endothelial cells of placental villi. The immunoexpression of JAM-C was elevated in PE vs. normotensive (N) placentae. In the exchange villi, JAM-C immunoexpression was higher in the N+ve vs. N-ve group. However, in PE comorbid HIV infection, JAM-C expression was lower in the PE+ve vs. PE-ve group. Citrullinated histone-H3 concentration was lower in the N+ve vs. N-ve group but elevated in early-onset PE (EOPE)+ve vs. late-onset PE (LOPE)+ve group. These results indicate that PE and HIV-infected placentae individually express elevated JAM-C, manifesting in less neutrophil r-TM. However, in exchange villi of PE comorbid with HIV infection reduced JAM-C enhances neutrophil r-TM, thus supporting the synergistic effect of PE comorbid with HIV.

## Introduction

Pre-eclampsia (PE) is characterized by new-onset hypertension with uteroplacental insufficiency and/or maternal symptoms, signs or abnormal laboratory tests (Magee et al. 2022). However, this definition is still evolving to facilitate the early detection of PE (Bartal and Sibai 2024). Sub-Saharan Africa is the epicenter of maternal deaths globally, constituting the leading region of human immunodeficiency virus (HIV) infection and the leading region of hypertensive disorders of pregnancy (HDP) (UNAIDS 2023; Ngene and Moodley 2024). One in four births in South Africa are from HIV-positive women, highlighting the importance of considering HIV status as a variable in elucidating maternal factors involved in the early detection of PE (Moyo et al. 2020).

The yin-yang effect of pro- and anti-inflammatory roles of PE and HIV respectively warrant the investigation of front runners of the immune system: neutrophils in these disorders (Moodley et al. 2020b). A key factor warranting the investigation of neutrophils is their ability to undergo reverse trans-migration (r-TM) from tissues back into the circulatory system where their pro-inflammatory state may produce NETs (Rizo-Téllez and Filep 2024; Xiao et al. 2023). Placental expression of junctional adhesion molecule C (JAM-C) is an inverse regulatory marker of neutrophil r-TM from the placenta (Cao et al. 2022; Woodfin et al. 2011). Junctional adhesion molecules (JAMs) are immunoglobulins that act at cellular and immunological levels by regulating epithelial and endothelial tight junction formation (Martìn-Padura et al. 1998) and permitting leukocyte and platelet transmigration (Muller 2003). Neutrophil r-TM is an inflammatory resolution process whereby neutrophils migrate out of tissues and re-enter the peripheral circulation (De Oliveira et al. 2016).

The initial detection of neutrophil r-TM was done in vitro using zebrafish larvae and indicated that most neutrophils leave the inflammatory site instead of via apoptosis (Mathias et al. 2006). These neutrophils travel the vasculature to distal sites (Yoo and Huttenlocher 2011; Hall et al. 2009). Notably, > 90% of neutrophils can reverse their direction and continually migrate for distances > 1000 μm (Hamza et al. 2014). Peripheral neutrophils in patients with systemic inflammation phenotypically express cell surface marker intercellular adhesion molecule-1 (ICAM1)^hi^ and chemokine receptor CXCR1^low^ (Buckley et al. 2006). The loss of JAM-C mediated tension on vascular endothelial cells by ischemia reperfusion injury promotes neutrophil r-TM (Yeh et al. 2020). Moreover, this inverse relationship between JAM-C expression and neutrophil r-TM is further amplified by increased neutrophil r-TM upon genetic deletion of JAM-C on endothelial cells (Weber et al. 2007). The resolution of neutrophil inflammation is also controlled by other processes such as neutrophil apoptosis with macrophage engulfment (Cox et al. 1995) and the formation of neutrophil extracellular traps (NETs) in a “death mechanism” named NETosis (Remijsen et al. 2011; Fuchs et al. 2007). However, the dysregulation of JAM-C in inflammatory resolution differs from these methods by causing dissemination of the inflammatory response to other systemic sites (Yoo and Huttenlocher 2011; Buckley et al. 2006; Loyer et al. 2022).

The production of reactive oxygen species (ROS) is a prerequisite to the formation of NETs (Brinkmann 2018). Reverse-migrated neutrophils have a stronger ROS response (Buckley et al. 2006). Leukotriene B4 induced enhanced expression of neutrophil elastase cleaves JAM-C, with a subsequent enhanced r-TM (Colom et al. 2015). Increased NETs are present in the pre-eclamptic placenta (Moodley et al. 2020b; Gupta et al. 2005). Along with the other neutrophil intracellular contents, elastase is also released in NETs (Brinkmann 2018). Therefore, this phenomenon implies that reduced JAM-C expression with a resultant concomitant increase in neutrophil reverse migration elevates neutrophil ROS production of NETs (Colom et al. 2015). During NETosis, neutrophils release NETs, a sticky chromatin mesh embedded with neutrophil cell-free DNA, granules and histones (Brinkmann 2018). Key steps of NETosis include neutrophil activation, reactive oxygen species (ROS) production and histone citrullination (Papayannopoulos 2018). Therefore, citrullinated histone H3 (cit-H3) is a specific marker of NETs (Thålin et al. 2017; Pan et al. 2017). As gestational age increases in pregnancy, there is a rise in circulatory neutrophils, with a concomitant reduction in neutrophil respiratory burst (Crocker et al. 2000). However, neutrophil dysregulation with enhanced NETosis predisposes subjects to PE (Gupta et al. 2005; Buckley et al. 2006). In PE, syncytiotrophoblasts (STB) secrete pro-inflammatory factors which produce elevated NETs (Gupta et al. 2005). During HIV infection, NETs are stimulated to ensnare and inactivate HIV; however, HIV has an evasion strategy which opposes NETs (Saitoh et al. 2012). Additionally, highly active antiretroviral therapy (HAART) has neutrophil and NET suppressive properties (Hadad et al. 2007). In the placenta, NETs are individually elevated in PE and HIV infection; however, NETs are synergistically downregulated in PE comorbid with HIV (Moodley et al. 2020b).

There is evidence of in vivo placental NET production in the dual interaction of PE and HIV infection (Moodley et al. 2020b), plasma NETs in HIV-naïve non-pregnant and pregnant (normotensive or pre-eclamptic) groups (Hu et al. 2018) and in vitro confirmation of NET release in HIV infection (Saitoh et al. 2012; Kozlowski et al. 2016). In the recent study reported on JAM-C in PE placentae, JAM-C was elevated in PE (Cao et al. 2022). However, HIV-infected PE placentae display a synergy response by suppressing the placental NET expression (Moodley et al. 2020b). During HIV infection, the trans-activator of transcription (Tat) protein contributes to endothelial injury by driving the expression of ICAM-1 and vascular cell adhesion protein-1 (V-CAM-1) (Liu et al. 2005). We hypothesize that the HIV-induced endothelial injury in PE results in reduced JAM-C expression, ultimately enhancing the movement of neutrophil r-TM from the placenta into the peripheral circulation. Therefore, the objectives were to immuno-localize and quantify JAM-C expression in placentae of normotensive (N) vs. pre-eclamptic (PE) women, additionally stratified by HIV status. Considering the paucity of information on serum NETs in HIV comorbid PE, this study aims to elucidate whether NETs may potentially be an effective marker of PE with or without HIV infection. Therefore, the additional objectives were to evaluate the concentration of cit-H3 as a specific marker of NETs in serum based on HIV status, pregnancy type (N vs. PE) and gestational age.

## Materials and methods

This study was conducted following ethical clearance; institutional ethics approval was obtained (BREC 764/2019) for the use of archived samples (BCA338/17). All women comprising the study participants attended a large regional hospital in KwaZulu-Natal Province, South Africa (SA), and provided written informed consent.

*Study population for the placental analysis***:** Pregnant women (*n* = 160) > 18 years old were recruited according to specific inclusion and exclusion criteria during their antenatal period at the Obstetric Unit of a large regional hospital in South Africa. The study groups were categorized based on pregnancy type [normotensive (*N*; *n* = 80) vs. pre-eclamptic (PE; *n* = 80)] and HIV status [HIV negative (HIV-ve; *n* = 80) vs. HIV positive (HIV+ve, *n* = 80)] and across the groups: [normotensive HIV negative (N-ve-; *n* = 40), PE HIV negative (PE-ve; *n* = 40), normotensive HIV positive (N+ve; *n* = 40) and PE HIV positive (PE+ve; *n* = 40)].

*Study population for the serum analysis***:** The total study population consisted of women (*n* = 80) who were normotensive non-pregnant (NNP) (*n* = 11) or pregnant (*n* = 69). The pregnant women were stratified by pregnancy type into normotensive (*N*; *n* = 29) or pre-eclampsia (PE; *n* = 40). These groups were further divided by HIV status into normotensive HIV negative (N-ve; *n* = 14), normotensive HIV+ve (N+ve; *n* = 15), pre-eclampsia HIV negative (PE-ve; *n* = 23) or pre-eclampsia HIV positive (PE+ve; *n* = 17). PE was further stratified by gestational age into early-onset PE HIV negative (EOPE-ve; *n* = 10), late-onset PE HIV negative (LOPE-ve; *n* = 13), early-onset PE HIV positive (EOPE+ve; *n* = 8) and late-onset PE HIV positive (LOPE+ve; *n* = 9).

Pre-eclampsia was characterized by new-onset hypertension, i.e, blood pressure > 140/90 mmHg on two separate occasions at least 4 h apart after 20 weeks gestation in the presence of proteinuria (urinary excretion of > 300 mg of protein in a 24-h urine collection or evidence of multi-organ involvement; Magee et al. 2022). EOPE was defined as the onset of PE at or after 20 weeks of gestation but prior to 33^+6^ weeks of gestation. LOPE was defined as the onset of PE at or after 34^+0^ weeks of gestation (Gomathy et al. 2018). All HIV+ patients were on highly active antiretroviral therapy (HAART).

### Placental sample collection and tissue preparation

Central regions of placental tissue were harvested, fixed, processed, embedded in paraffin wax, sectioned at 5 microns (μm) and collected onto charged slides as previously described (Onyangunga et al. 2017; Cao et al. 2022).

### Immunohistochemistry

**Fig. 1:**
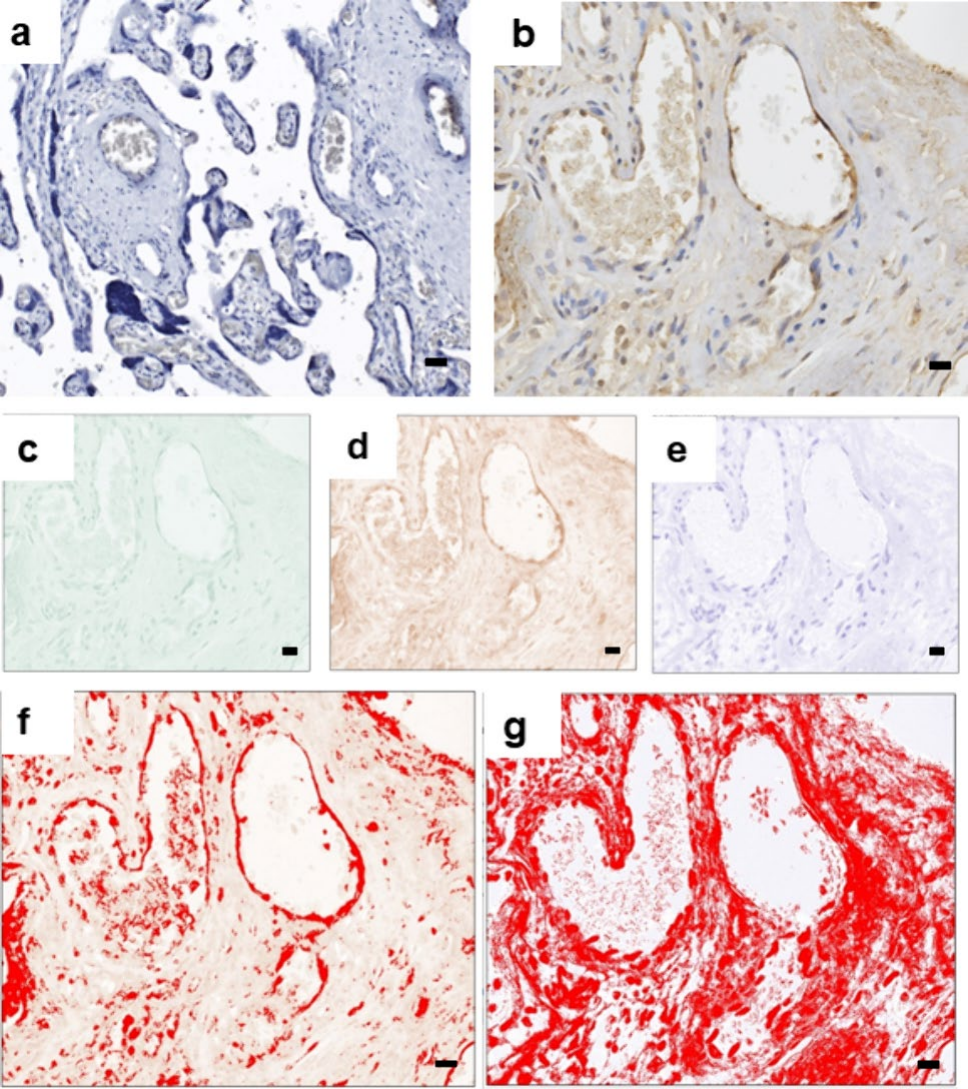
Immunohistochemistry JAM-C controls and morphometric image analysis. **a** Placenta negative control and **b** placenta positive control tissues are represented. Quantification of JAM-C expression was performed **c–g** for each field of view. **b** Placental villi field of view was opened in Image J software and underwent color deconvolution into the **c** green channel, **d** red channel and **e** blue channel. **f** Threshold of chromogen (brown stain) in the red channel was calculated as a percentage expression of JAM-C in the frame and **g** threshold expression of hematoxylin (blue) was calculated as a percentage expression of villi area in the frame. Magnification × 40, scale bar lengths 20 μm

Immunohistochemistry was performed using the rabbit-specific HRP/DAB (ABC) detection IHC kit (ab64261; Abcam, UK). Following heat-mediated antigen retrieval with citrate buffer pH 6 (Onyangunga et al. 2017), the placental tissue was incubated with the anti-JAM-C primary antibody (ab2243327, Abcam, UK) to immunolabel JAM-C+ (1/20 dilution; overnight at 4 °C). The samples were incubated with the chromogen, counterstained and cover slipped as described previously (Onyangunga et al. 2017). Replacement of the primary antibody with a buffer and non-immune sera of the same IgG class served as the negative control placental tissue (Fig. 1A). The positive control placental tissue was stained with the anti-JAM-C primary antibody (Fig. 1B).

### Morphometric image analysis

Zeiss Apotome Imaging System (Carl Zeiss, Germany) and Zen blue software (Carl Zeiss, Germany) were used to examine and capture placental tissue respectively at an initial objective magnification of × 40 within four fields of view. Quantification of JAM-C expression was performed for each field of view. The placental villi field of view was opened in Image J software [Fiji version, National Institute of Health (NIH), Wisconsin, USA] and underwent color deconvolution into the green channel (Fig. 1C), red channel (Fig. 1D), and blue channel (Fig. 1D). The threshold of chromogen (brown stain) in the red channel was calculated as a percentage expression of JAM-C in the frame, and the threshold expression of hematoxylin (blue stain) in the blue channel was calculated as a percentage expression of villi area in the frame (Fig. 1 F-G). The percentage expression of JAM-C per villi area in the frame was calculated as [%brown/(%brown + %blue) × 100]. The immuno-reactivity was expressed as a percentage of JAM-C label within the conducting villi and exchange villi, respectively.

### ELISA reagents and equipment

Serum cit-H3 was quantified using the Citrullinated Histone H3 (Clone 11D3) ELISA Kit (Cayman Chemical, Ann Arbor,MI, USA, item no. 501620). This kit contained an anti-histone H3 HRP conjugate, anti-Citrullinated Histone H3 ELISA Strip Plate, Citrullinated Histone ELISA Standard, Immunoassay Buffer B Concentrate (10×), wash buffer concentrate (400×), polysorbate 20, TMB substrate solution, HRP Stop solution and a 96-well cover sheet. A Heidolph shaker (Labotech, Midrand, South Africa) was used. A Nano SPECTROstar plate reader (BMG Labtech, Ortenberg, Germany) interfaced with appropriate software was used to read absorbance and to analyze the data.

### ELISA methodology

Briefly, 100 μl of the standards or diluted samples were added to the wells of the 96-well plate. The plate was covered and incubated for 2 h at room temperature on an orbital shaker. The wells were then emptied and samples placed in wash buffer. Thereafter, 100 μl of HRP Conjugate Working Solution was added to each well. The plate was covered again, and the second incubation took place on an orbital shaker for 1 h. The wells were washed, and 100 μl of TMB Substrate Solution was added to each well. The third incubation of the plate occurred in the dark on the orbital shaker for 30 min. Next, 100 μl of HRP Stop Solution was added to each well. The plate was read at a wavelength of 450 nm on a plate reader. The ELISA assay was run in duplicate with a measurement range of 0.15–10 ng/ml. Measurements of standards and samples were done against the test sample blank at a wavelength of 450 nm. The standard curve was plotted using the concentration of cit-H3 (ng/ml) against absorbance (450 nm).

### Statistical analysis

GraphPad Prism (version 8.0.2) statistical software (San Diego, CA, USA) was used to analyze the results. The one-way analysis of variance (ANOVA) test was used for comparing three or more independent groups of normally distributed data. Kolmogorov-Smirnov normality test was used to determine the distribution of the data. Student’s unpaired *t*-test was used for dual-group analyses of parametric data (represented as the mean and standard deviation), and the Mann-Whitney U test was used for non-parametric data (represented as the median and interquartile range). Pearson’s r ranked correlations were used since correlation data were parametrically distributed. *P*  < 0.05 was considered statistically significant.

## Results

### Patient demographics and clinical data

The patient demographics and clinical data for the patients of the placenta samples are represented (Table 1). As expected, pre-eclampsia has high systolic (*p* < 0.0001) and diastolic blood pressure (*p* < 0.0001) compared to normotensive women. In pre-eclampsia, the gestational ages are lower than in normotensive women (*p* = 0.0015). The CD4 + T cell count is higher in the PE+ve group compared to the N+ve group (*p* = 0.0184).

**Table 1:** Patient demographics and clinical data for the placenta analysis

Clinical characteristics	^a^N-ve	^b^PE-ve	^c^N+ve	^d^PE+ve	*p* value
Maternal age (years)	23.59 ± 5.22	26.00 ± 6.96	27.03 ± 4.76	30.16 ± 5.86	*p* = 0.0004***
Gestational age (weeks)	38.53 ± 1.04	37.73 ± 2.95	38.98 ± 1.45	37.00 ± 2.56	*p* = 0.0015**
Maternal weight (kg)	68.58 ± 10.58	79.53 ± 18.43	76.11 ± 10.56	85.87 ± 24.16	*p* = 0.0069**
Systolic pressure (mmHg)	114.8 ± 10.66	156.6 ± 13.92	113.5 ± 10.55	152.6 ± 9.03	*p* < 0.0001****
Diastolic pressure (mmHg)	70.59 ± 6.85	94.37 ± 12.76	70.00 ± 6.78	92.40 ± 9.67	*p* < 0.0001****
CD4 + T cell count (cells/μl)	–	–	273.5 (IQR = 172.1)	352.0 (IQR = 239.5)	*p* = 0.0184*
Birth weight (kg)	3.17 ± 0.36	3.17 ± 0.78	3.41 ± 0.48	2.99 ± 0.57	*p* = 0.0358*

Significant *p* values of the one-way ANOVA are illustrated. The means ± standard deviation of each subgroup are represented; median and IQR are represented for the CD4 + T cell counts

*P* < 0.05 is denoted as statistically significant. Levels of significance are as follows: *p < 0.05, **p < 0.01, ***p < 0.001 and ****p < 0.0001

^a^N-ve: normotensive HIV negative

^b^PE-ve: pre-eclampsia HIV negative

^C^N+ve: normotensive HIV positive

^d^PE+ve: pre-eclampsia HIV positive

Using the one-way analysis of variance (ANOVA) test, significant differences were detected across the study population from which the maternal serum was collected for the following parameters: maternal age (*p* = 0.0016), gestational age (*p* = 0.0019), systolic blood pressure (*p* < 0.0001), diastolic blood pressure (*p* < 0.001) and baby birth weight (*p* = 0.0042) (Table 2).

**Table 2:** Patient demographics and clinical data for the serum neutrophil extracellular traps analysis

Clinical characteristics	^a^NNP+ve	^b^N-ve	^c^N+ve	^d^PE-ve	^e^PE+ve	*p* value
Maternal age (years)	32.91 ± 7.20	25.14 ± 6.10	25.00 ± 3.70	24.88 ± 5.27	27.33 ± 4.11	*p* = 0.0016**
Gestational age (weeks)	–	39.07 ± 1.85	38.73 ± 1.83	34.64 ± 5.36	34.90 ± 4.63	*p* = 0.0019 **
Maternal weight (kg)	72.62 ± 16.45	78.36 ± 14.50	72.08 ± 15.55	72.08 ± 14.99	83.61 ± 19.25	*p* = 0.2319
Body mass index (BMI) (kg/m2)	30.18 ± 4.75	31.36 ± 5.41	26.40 ± 8.96	28.59 ± 3.51	30.42 ± 12.21	*p* = 0.4283
Systolic pressure (mmHg)	122.30 ± 23.64	119.60 ± 7.96	122.00 ± 10.70	164.90 ± 17.04	161.1 ± 25.21	*p* < 0.0001***
Diastolic pressure (mmHg)	73.00 ± 21.02	72.29 ± 8.96	75.13 ± 9.28	99.47 ± 13.01	98.83 ± 17.83	*p* < 0.0001***
CD4 + T cell count (cells/μl)	500 (IQR = 387)	–	391 (IQR = 199.8)	–	413 (IQR = 334)	*p* = 0.8306
Baby weight (kg)	n/a	3.303 ± 0.38	3.271 ± 0.38	2.73 ± 1.03	2.52 ± 1.01	*p* = 0.0042**

Significant *p* values of one-way ANOVA are illustrated. Means ± the standard deviation of each subgroup are represented; median and IQR are represented for the CD4 + T cell counts

*P* < 0.05 is denoted as statistically significant. Levels of significance are as follows: *p < 0.05, **p < 0.01, ***p < 0.001

^a^NNP+ve: normotensive non-pregnant HIV positive

^b^N-: normotensive HIV negative

^c^N +: normotensive HIV positive

^d^PE-: pre-eclampsia HIV negative

^e^PE +: pre-eclampsia HIV positive

### Immuno-localization of junctional adhesion molecule C and histological features of placental villi

**Fig. 2:**
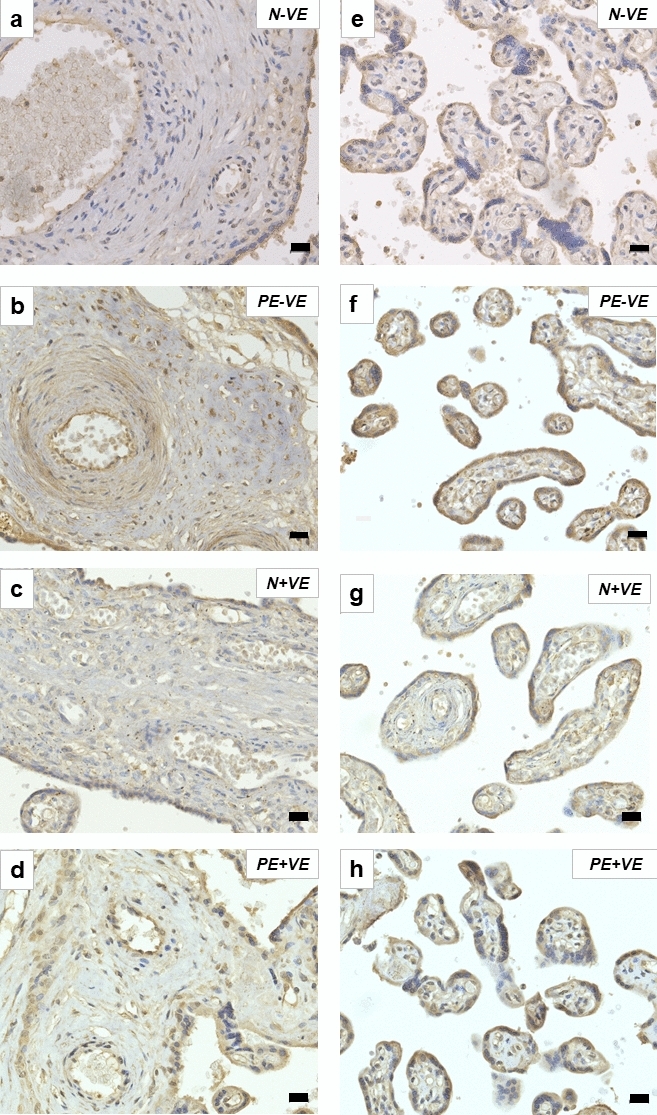
Immuno-localization of JAM-C in the placental villi. JAM-C was expressed on the conducting villi **a–d** and exchange villi **e–h**. In all groups and villi types, JAM-C was expressed on syncytiotrophoblasts (STB) and on the endothelium (EN) which surrounded the red blood cells (RBC) and circulatory cells of arteries, veins and capillaries. Smooth muscle (SM) cells expressed JAM-C. Additionally, JAM-C was expressed on neutrophils (NE), particularly in their cytoplasm. The highest JAM-C expression was observed in the PE-ve group in both the conducting villi **b** and exchange villi **f**. Magnification ×40, scale bar lengths 20 μm

**Fig. 3:**
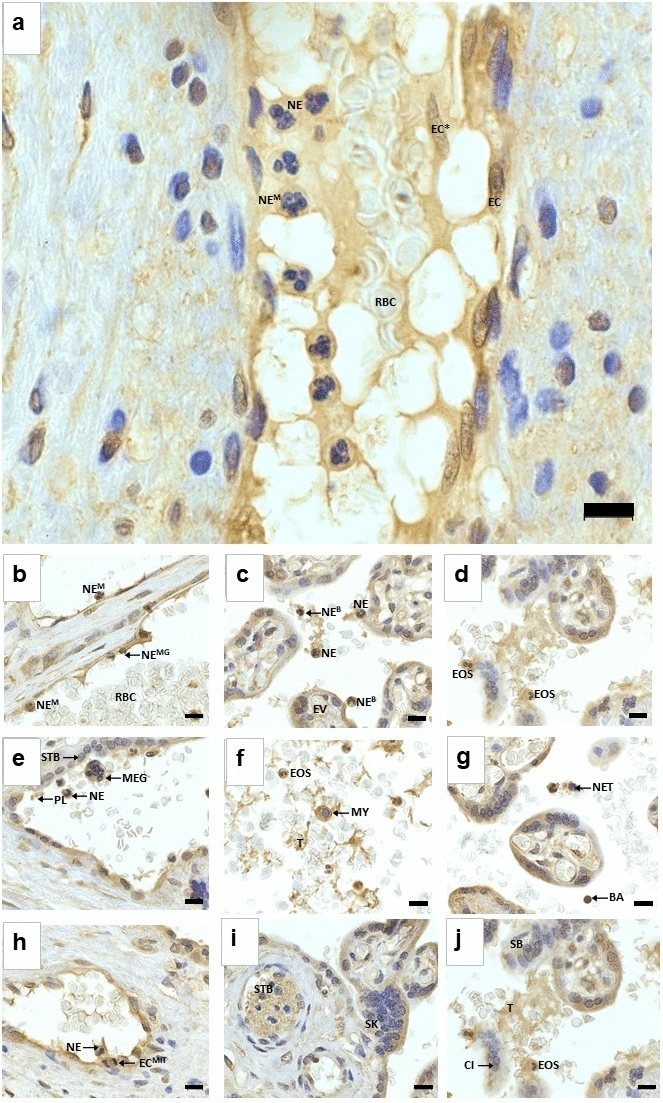
Histology and unique features of JAM-C expression in the placenta at 100× magnification. Key: *BA* basophil; *CI* cell island; *EC* endothelial cell; EC* endothelial cell sloughed off in circulation; *EC*^*MIT*^ mitotic endothelial cell; *EOS* eosinophil; *EV* exchange villi; *MEG* megakaryocyte; *MY* myeloblast; *NE* neutrophil; *NE*^*B*^ neutrophil band cell; *NE*^*M*^ mature neutrophil, *NE*^*MG*^ neutrophil migration; *PL* platelet; *SB* syncytial bridge; *SK* syncytial knot; *STB* syncytiotrophoblast; *T* thrombin. **a** Positive staining for JAM-C was observed in the cytoplasm of neutrophils (NE). Some of the neutrophils are adhering to the endothelium of the vessel, and the other neutrophils appear to be undergoing chemotaxis. Some of the neutrophils are mature (NE^M^) with five lobes. An endothelial cell has sloughed off and is in the circulation (EC*). **b** Migrating neutrophils (NE^MG^) are observed, and mature neutrophils are attached to the endothelium. **c** In the intervillous space surrounding the exchange villi (EV), neutrophils are observed in their immature state as a neutrophil band cell (NE^B^). Both band cells and neutrophils are observed attached to the syncytiotrophoblast epithelium. **d** Cytoplasm of eosinophils (EOS) was positively stained for JAM-C. **e** Megakaryocyte (MEG) cytoplasm is positively stained for JAM-C. Platelets also express JAM-C (PL), and the cytoplasm of syncytiotrophoblasts (STB) express JAM-C. **f** In the intervillous space, the cytoplasm of the myelocyte (MY) was positively stained for JAM-C. Eosinophils are also present, and thrombin was positively stained for JAM-C. **g** Basophils (BA) are JAM-C positive, and neutrophil extracellular traps (NETs) express JAM-C. **h** A neutrophil is observed adhered to the endothelium, and a mitotic endothelial cell (EC^MIT^) is seen. **i** A syncytial knot (SK) is observed. **j** A syncytial bridge (SB) and cell island (CI) are present, with EOS and T nearby. Magnification ×100, scale bar lengths 10 μm

Junctional adhesion molecule-C was localized on the syncytiotrophoblasts (STB) and endothelial cells (EC) in the conducting and exchange villi (Fig. 2). Qualitatively, JAM-C immunoexpression appeared to be higher in pre-eclamptic compared to normotensive women in both the conducting (Fig. 2A–B) and exchange villi (Fig. 2E-F). On higher magnification, JAM-C expression was localized on syncytial knots, syncytial bridges and cell islands (Fig. 3). Thrombin showed positive JAM-C expression in the intervillous space. JAM-C was also immunoexpressed on many blood cell types such as neutrophils (including neutrophil band cells and mature neutrophils), eosinophils, megakaryocytes, platelets, myeloblasts and basophils (Fig. 3).

### Quantitation of JAM-C placental villi expression using morphometric image analysis

#### Conducting villi

**Fig. 4:**
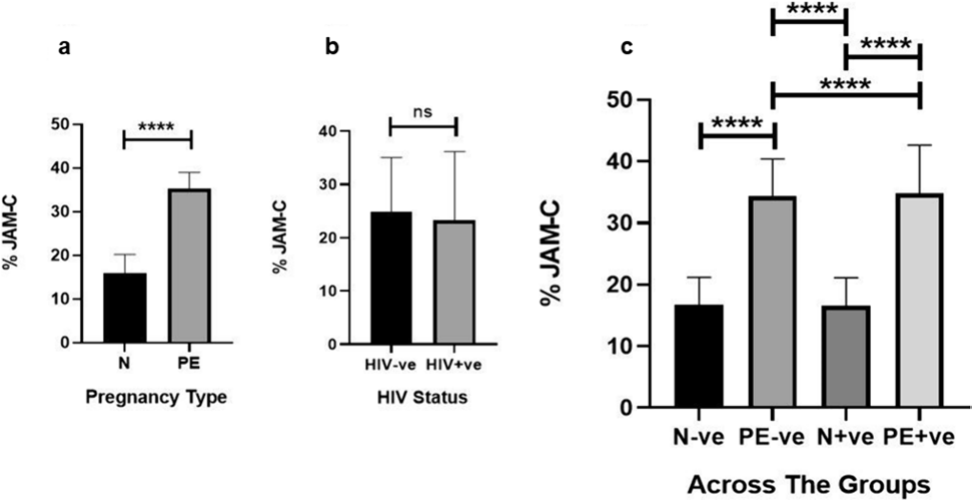
Placental conducting villi percentage (%) JAM-C expression between pregnancy type and HIV status and across the sub-populations. **a** %JAM-C expression was higher in PE compared to the N group. **b** There was no statistical difference in the %JAM-C expression between the HIV-ve vs. HIV+ve groups. **c** %JAM-C expression was higher in the PE-ve compared to the N-ve group. The PE+ve group had higher %JAM-C expression than the PE-ve group and compared to the N+ve group. The PE-ve group had higher %JAM-C expression than the N+ve group

According to pregnancy type and irrespective of HIV status, the immunoexpression of JAM-C was higher in PE compared to the N group [15.96% (IQR = 6.28) vs. 35.26% (IQR = 9.70), *p* < 0.0001] (Fig. 4A). According to HIV status and regardless of pregnancy type, there was no significant difference in JAM-C expression [24.84% (IQR = 18.40) vs. 23.21% (IQR = 20.36), *p* = 0.9661] (Fig. 4B). Across the groups, JAM-C expression was higher in the PE-ve compared to the N-ve group (34.35% ± 6.04 vs. 16.70% ± 4.48, *p* < 0.0001), PE+ve compared to the N+ve group (34.85% ± 67.80 vs. 16.58% ± 4.53, *p* < 0.0001), PE+ve compared to the N-ve group (34.85% ± 67.80 vs. 16.70% ± 4.48, *p* < 0.0001) and the PE-ve group compared to the N+ve group (34.35% ± 6.04 vs. 16.58% ± 4.53, *p* < 0.0001). There was no significant difference in JAM-C expression between the N-ve compared to the N+ve groups (16.70% ± 4.48 vs. 16.58% ± 4.53, *p* = 0.9036) (Fig. 4C).

#### Exchange villi

**Fig. 5:**
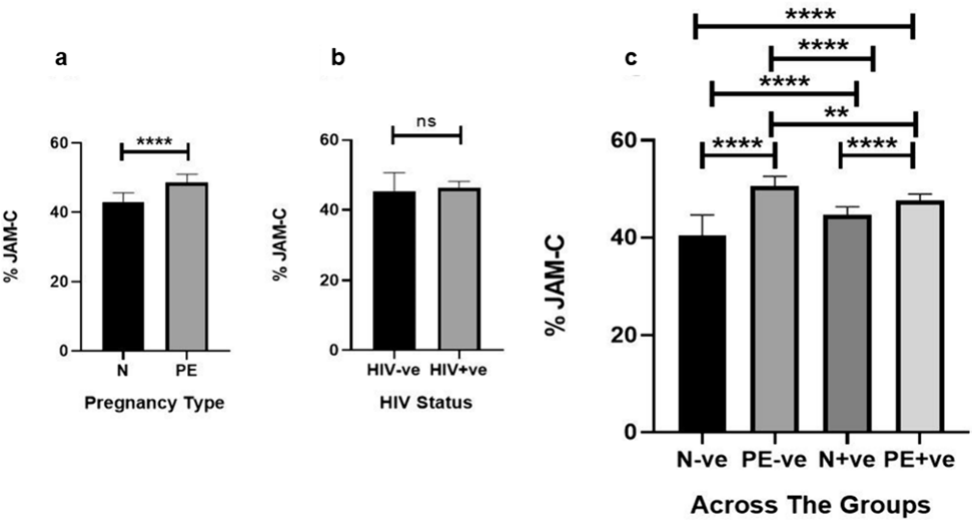
Placental exchange villi percentage (%) JAM-C expression between pregnancy type, HIV status and across the sub-populations.** a** %JAM-C expression was higher in PE compared to the N group. **b** There was no statistical difference in the %JAM-C expression between the HIV-ve vs. HIV+ve groups. **c** %JAM-C expression was higher in the PE-ve compared to the N-ve group. PE+ve group had lower %JAM-C expression than the PE-ve group. The PE+ve group had higher %JAM-C expression compared to the N+ve group. The PE-ve group had higher %JAM-C expression compared to the N+ve group and compared to the N-ve group. The N+ve group had higher %JAM-C expression than the N-ve group, but the N+ve group had lower %JAM-C expression than the PE-ve group

According to pregnancy type and irrespective of HIV status, the expression of JAM-C was higher in the PE compared to the N group [48.60% (IQR = 4.77) vs. 43.08% (IQR = 7.14), *p* < 0.0001] (Fig. 5A). According to HIV status and irrespective of pregnancy type, JAM-C expression was not statistically different between the HIV-ve *vs*. HIV+ve groups [45.33% (IQR = 10.41) vs. 46.26% (IQR = 4.30), *p* = 0.4863] (Fig. 5B). Across the groups in HIV-ve placentae, JAM-C expression was higher in the PE-ve compared to N-ve group [50.64% (IQR = 6.01) vs. 40.39% (IQR = 9.28), *p* < 0.0001], the PE+ve compared to N+ve group [47.69% (IQR = 2.77) vs. 44.61% (IQR = 3.70), *p* < 0.0001], the PE+ve group compared to the N-ve group [47.69% (IQR = 2.77) vs. 40.39% (IQR = 9.28), *p* < 0.0001], the PE-ve group compared to the N+ve group [50.64% (IQR = 6.01) vs. 44.61% (IQR = 3.70), *p* < 0.0001] and the N+ve compared to the N-ve group [44.61% (IQR = 3.70) vs. 40.39% (IQR = 9.28), *p* < 0.0001]. JAM-C expression was lower in the PE+ve compared to the PE-ve group [47.69% (IQR = 2.77) vs. 50.64% (IQR = 6.01), *p* = 0.0025] (Fig. 5C).

### Serum cit-H3 concentration analysis

#### Serum cit-H3 concentrations across pregnancy type and HIV status

**Fig. 6:**
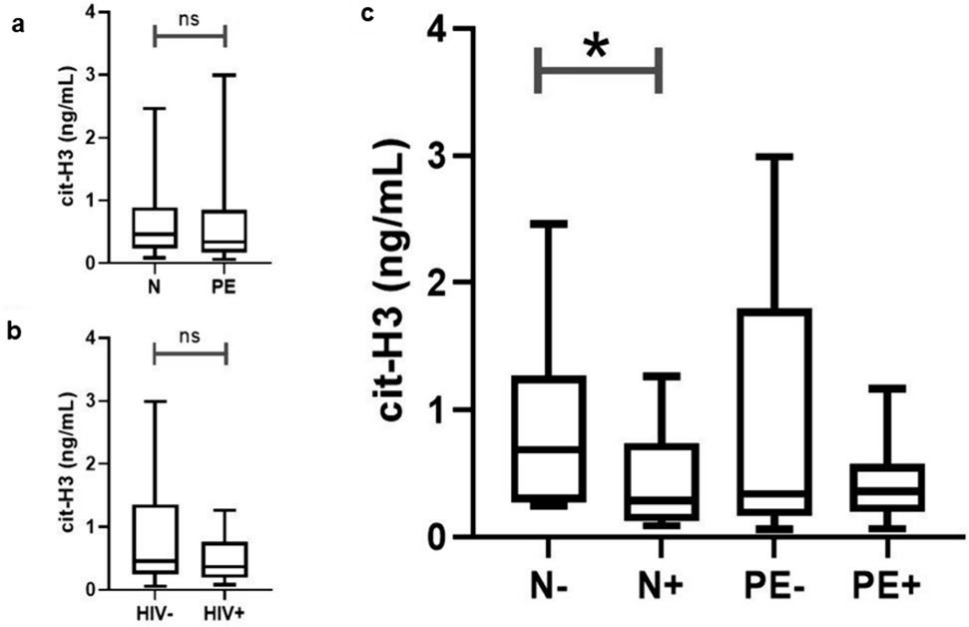
Serum cit-H3 concentrations between pregnancy type and HIV status and across the sub-populations. **a** There was no significant difference in cit-H3 concentration between N vs. PE and **b** between the HIV-ve vs. HIV+ve groups. **c** Concentration of serum cit-H3 was lower in the N+ve compared to the N-ve group

Pregnancy type had no statistically significant effect on serum cit-H3 concentration regardless of HIV status (*p* = 0.5437) (Fig. 6A). Irrespective of pregnancy type, the concentration of cit-H3 was also not statistically different between the HIV+ve group compared to the HIV-ve controls (*p* = 0.2846) (Fig. 6B).

#### Serum cit-H3 concentration across the subgroups of the study population

The concentration of serum cit-H3 was significantly downregulated in the N+ve compared to N-ve group [0.2850 ng/ml (IQR = 0.6200) vs. 0.6885 ng/ml (IQR = 1.0000), *p* = 0.0259] (Fig. 6C). In PE, there was no difference in cit-H3 concentration between the PE-ve and PE+ve groups (*p* = 0.6849) (Fig. 6C).

#### Serum cit-H3 concentration across gestational age

**Fig. 7:**
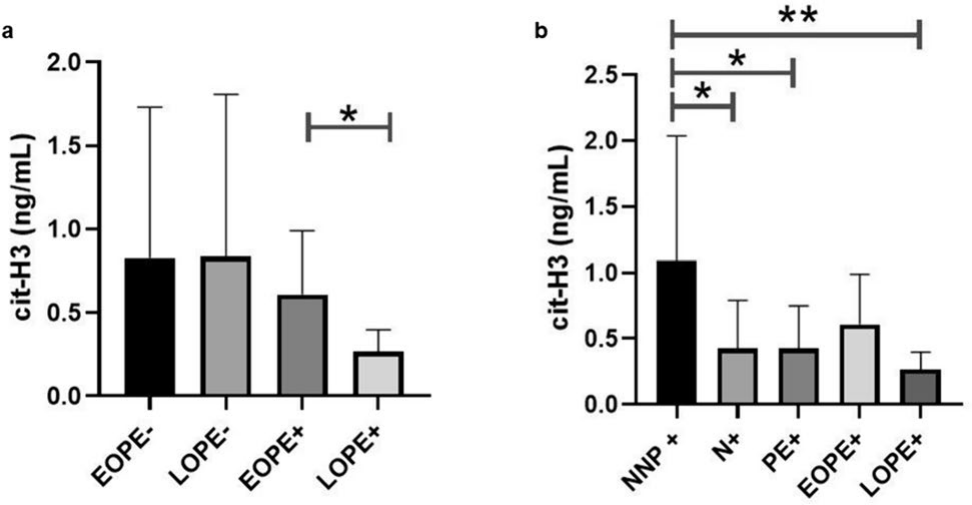
Serum cit-H3 concentrations between the onset of pre-eclampsia with respective HIV status and between non-pregnant and pregnant HIV-positive subgroups. Cit-H3 concentration in **a** LOPE+ve was compared to the EOPE+ve group; **b** pregnant N+ve, PE+ve and LOPE+ve groups were compared to the NNP+ve group

The concentration of cit-H3 was elevated in EOPE+ve compared to LOPE+ve group (0.6095 ng/ml ± 0.3801 vs. 0.2667 ng/ml ± 0.1331, *p* = 0.0224) (Fig. 7.A). In the HIV-ve group, there was no statistical difference in cit-H3 concentration between the EOPE- and LOPE- groups.

#### Serum cit-H3 concentration between HIV positive non-pregnant and pregnant subgroups

As an explorative aspect of the study, we compared serum cit-H3 expression as a marker of NETs between non-pregnant and pregnant subgroups (Fig. 7B). In the NNP+ve group, the concentration of cit-H3 was elevated compared to the pregnant N+ve group [0.7450 ng/ml (IQR = 0.9910) vs. 0.2850 ng/ml (IQR = 0.6200), *p* = 0.0150]. The NNP+ve group also had higher cit-H3 concentration compared to the PE+ve group [1.0910 ng/ml ± 0.9451 vs. 0.4280 ± ng/ml 0.3212, *p* = 0.0125], and cit-H3 concentration was also higher in the NNP+ve compared to the LOPE+ve group (1.0910 ng/ml ± 0.9451 vs. 0.2667 ng/ml ± 0.1331, *p* = 0.0188) (Fig. 7B). However, there was no statistical difference in cit-H3 concentration between the NNP + and EOPE + groups.

#### Correlations between serum cit-H3 concentration and maternal clinical data

**Fig. 8:**
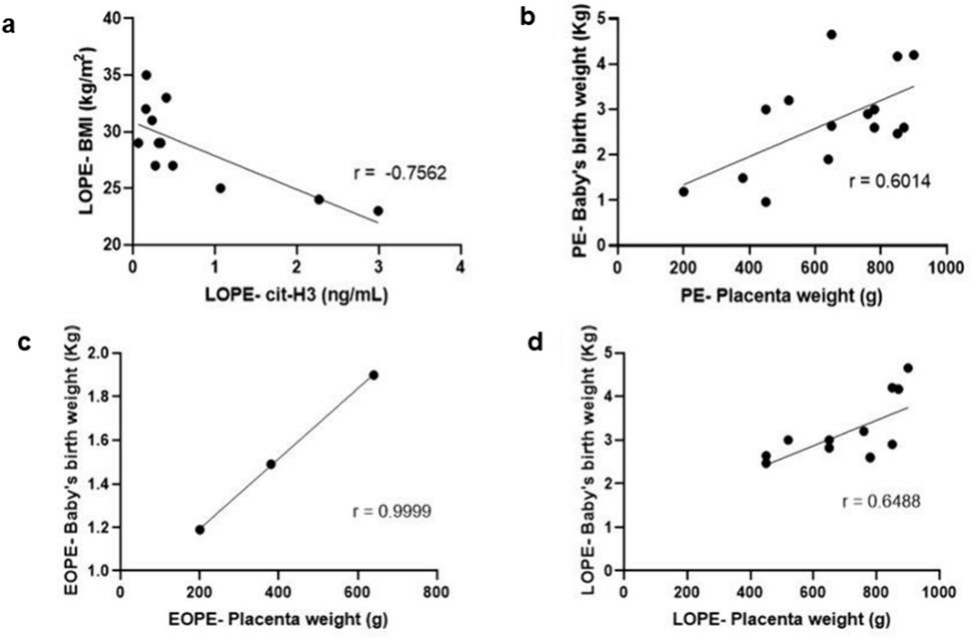
Significant correlations of cit-H3 and clinical data. **a** Mother’s body mass index [BMI (kg/m^2^)] in the LOPE-ve group was inversely correlated to cit-H3 concentration. Placenta weight inversely correlated with baby’s birth weight in the **b** PE-ve group, **c** EOPE-ve group and **d** LOPE-ve group

Serum cit-H3 concentration was inversely related to mother’s BMI in the LOPE-ve group (*r* =− 0.7562, *p* = 0.0044) (Fig. 8A). There were positive correlations between baby’s birth weight and placenta weight in the PE-ve group (*r* = 0.6014, *p* = 0.0177) (Fig. 8B), EOPE-ve group (*r* = 0.9999, *p* = 0.0098) (Fig. 8C) and LOPE-ve group (*r* = 0.6488, *p* = 0.0225) (Fig. 8D).

## Discussion

### Summary

The pivotal role of JAM-C in placental-maternal regulation is validated by the multifaceted expression of JAM-C in both placental villi (syncytiotrophoblast and endothelial cells) and within immune cells. Individually, PE and HIV show enhanced JAM-C expression, thus preventing neutrophil r-TM. However, in the exchange villi, PE comorbid with HIV has a synergistic response by suppressing JAM-C expression, thus enhancing neutrophil r-TM.

This study demonstrates a reduction of serum NETs in N+ve pregnant compared to N-ve pregnant women. We report no significant difference in the level of circulating NETs between N and PE pregnant women. However, NETs were elevated in EOPE+ve compared to LOPE+ve groups. Additionally, an elevation of serum NETs was observed in NNP+ve compared to N+ve, PE+ve and LOPE+ve subgroups. Lastly, in the LOPE-ve group, serum NETs were inversely correlated with maternal BMI.

### Junctional adhesion molecule C expression as a marker of neutrophil r-TM

This study is novel in that it immuno-localizes and morphometrically quantifies JAM-C expression in N vs. PE placentae were stratified by HIV status and villi type in conducting villi and exchange villi. The main findings are that individually PE and HIV had elevated JAM-C expression. However, PE comorbid HIV synergistically downregulated JAM-C expression. Resultantly, the enhanced neutrophil migration may potentially be an effective marker of PE in HIV-infected women.

Immunostaining of JAM-C was observed across all study groups in both the conducting and exchange villi. Overall, across all study groups, JAM-C was expressed on syncytiotrophoblast cells of the conducting and exchange villi and on endothelial cells of arteries, veins and capillaries. A recent study reported JAM-C immunolocalization and quantification in N vs. PE placentae (Cao et al. 2022). Syncytiotrophoblasts expressed JAM-C; however, JAM-C was scarcely observed in normotensive HIV-ve placentae, similar to reports by Cao et al. (2022). In normotensive term placentae, more CTBs differentiate into STBs, with a resultant reduction in JAM-C expression (Cao et al. 2022). In first trimester placentae, JAM-C is expressed on the cell membrane of CTB progenitors in anchoring villi, and JAM-C expression decreases as gestational age increases (Cao et al. 2022). This decrease in JAM-C expression with increasing gestational age is due to the trophoblasts acquiring invasion properties at 8–10 weeks gestation (wave 1) and 18–18 weeks gestation (wave 2) (Cao et al. 2022; Lyall et al. 2001). The expression of JAM-C is relatively reduced in second compared to first trimester placentae, and JAM-C expression is decreased further in third trimester placentae (Cao et al. 2022). In HIV+ve normotensive placentae and HIV+ve pre-eclamptic placentae, JAM-C was localized on STBs and endothelial cells. This study also reports the localization of JAM-C in immune system components: neutrophils [3-lobe, 5-lobe (mature neutrophils), neutrophil band cells and neutrophil extracellular traps], myeloblasts, megakaryocytes, platelets, eosinophils and basophils.

### Quantification of placental junctional adhesion molecule C expression

#### Conducting villi

In the conducting villi, JAM-C expression was elevated in pre-eclamptic placentae compared to normotensive placentae of mixed HIV status. When controlling for HIV status, JAM-C expression remained higher in PE compared to N groups in N-ve vs. PE-ve and N+ve vs. PE+ve groups. This study is novel in reporting the elevation of JAM-C in PE compared to N groups in HIV+ve placentae. In PE, an elevation of JAM-C contributes to the pathogenesis by: (1) suppressing the differentiation of CTBs into extravillous trophoblasts and (2) inhibiting the migration and invasion of extravillous trophoblasts through the β-catherin signaling pathway. Additionally, a JAM-C-infected murine model mimicked the human PE phenotype (Cao et al. 2022). In PE, there is an immense upregulation of JAM-C on STBs of floating villi and extravillous trophoblasts of the placental basal plates (Cao et al. 2022).

The quantification of JAM-C in HIV-ve and HIV+ve placentae was similar. When controlling for pregnancy type, JAM-C expression was also similar in N-ve vs. N+ve and PE-ve vs. PE+ve groups. Therefore, HIV had no effect on JAM-C within the conducting villi.

#### Exchange villi

In the exchange villi, JAM-C expression was elevated in pre-eclamptic placentae compared to normotensive placentae. When controlling for HIV status, JAM-C expression remained higher in PE compared to N groups in N-ve vs. PE-ve and N+ve vs. PE+ve groups. This is similar to our observations in the conducting villi and similar to HIV-ve reports by Cao et al. (2022).

When comparing HIV status, JAM-C expression was similar in the exchange villi. However, when controlling for pregnancy type in the exchange villi, JAM-C expression was elevated in N+ve compared to N-ve placentae. This is a novel study reporting that HIV-infected placentae have higher JAM-C expression in the exchange villi compared to HIV naïve placentae.

### Junctional adhesion molecule C and neutrophil reverse transmigration in pre-eclampsia comorbid human immunodeficiency virus infection: defying the expected

Since PE and HIV infection both individually culminate in elevated JAM-C expression, we anticipated that PE+ve placentae would have higher JAM-C expression compared to PE-ve placentae. However, to our surprise we noted a decline in JAM-C expression within the exchange villi in HIV-infected PE placentae compared to HIV-naïve PE placentae. Similarly, we reported an individual elevation of NETs in PE and HIV infection, with a contrasting suppression in NETs in PE+ve compared to N+ve placentae (Moodley et al. 2020b). Neutrophils carry inflammatory signals from tissues, back into the circulation, to other distal sites in a process of neutrophil r-TM. The expression and comparison of JAM-C among the placental subgroups of this study are similar to comparison of placental NETs (Moodley et al. 2020b), strengthening the link between JAM-C expression and neutrophil r-TM. It is unclear at this stage in which direction the cause and effect may occur in terms of suppressed NETs and reduced JAM-C expression in the duality of PE and HIV in the placenta. The exchange villi are the primary site of the regulated exchange of substances between the mother and baby. Less JAM-C expression in the exchange villi in the duality of PE and HIV translates to more neutrophils being able to transmigrate back into the maternal circulation, carrying pro-inflammatory signals and potentially harming the mother. Therefore, it is plausible that the suppressed NETs in PE comorbid HIV infection is a compensatory protective mechanism to counteract the maternal neutrophil overload resultant of placental neutrophil r-TM into the maternal circulation. In the exchange villi, JAM-C quantificational expression and comparison are inverse to the expression of receptor-expressing histone 2A (H2A)-positive STBs in all groups (N vs. PE, N-ve vs. N+ve, N-ve vs. PE-ve) except for the N+ve vs. PE+ve group where JAM-C expression is directly related with the expression of receptor-expressing H2A positive STBs. This pattern is similarly observed in JAM-C and receptor-less H2A negative STBs (Moodley et al. 2020a).

### Junctional adhesion molecule C specificity and its role in neutrophil reverse migration

Tissue integrity is regulated by cell-cell interactions and epithelial and endothelial barriers between different tissues and the circulation. These barriers mediate the regulation of inflammatory cell transmigration and defend against toxic or infective agents. On the barriers, adjacent cells express adhesion receptors which facilitate inter- and intracellular signals. Junctional adhesion molecules are expressed on tight junctions of epithelial and endothelial cells and function in regulating tight junction formation, permeability and leukocyte transmigration (Keiper et al. 2005). In addition to the chemokine signaling in leukocyte transmigration and diapedesis, there is also mechanical involvement. Leukocytes exert contractile stresses on endothelial cells which facilitate the opening of tight junctions. They then generate strong stresses and push themselves through the gap into the matrix (Yeh et al. 2018). JAM-C between endothelial cells is a potent neutrophil r-TM regulator, and the biochemical mechanisms of JAM-C are involved in regulating the contractility of the actin-myosin layer of vascular endothelial cells (VECs). Additionally, the knockdown of JAM-C led to decreased monolayer stress and tension of VECs and promoted increased r-TM of neutrophils (Yeh et al. 2020). Moreover, in vivo studies show that decreased expression of JAM-C at endothelial junctions leads to disrupted polarized forward neutrophil migration and enhanced neutrophil r-TM (Woodfin et al. 2011). The specificity of JAM-C in regulating polarized neutrophil migration is strengthened by the inability of JAM-A to significantly affect this (Woodfin et al. 2011).

### Maternal serum citrullinated histone H3 expression as a specific marker of neutrophil extracellular traps

#### Neutrophil extracellular traps are downregulated in human immunodeficiency virus-infected sera

We report a reduction of serum NETs in N+ve pregnant women compared to N-ve pregnant women. During an HIV infection, NETs are released to trap and inactivate the virus (Saitoh et al. 2012; Kozlowski et al. 2016; Barr et al. 2018) . However, previous studies show that HIV infection is linked to neutropenia (Levine et al. 2006; Kourtis et al. 2012) making it plausible that, although HIV infection is NET-stimulatory, the overall neutropenia reduces serum NETs in HIV+ve pregnant women. Also, HIV has developed evasive strategies to eradicate NETs by acting on dendritic cells to promote the release of anti-inflammatory interleukin 10 (IL-10) (Saitoh et al. 2012). In this study, all HIV+ women received HAART, a standard of care therapy to decrease viral load; this ultimately reduces HIV-mediated formation of NETs. Additionally, HAART has anti-stimulatory properties on neutrophils by causing neutropenia (Bello 2016), enhancing neutrophil chemotaxis to tissues (Moore et al. 1998) and inhibiting ROS (Hadad et al. 2007). Thus, the reduction of NET release in HIV infection is exacerbated by the neutropenia effect of HAART, which further aggravates the downregulation of NET production.

Interestingly, our results are in contrast to the expression of NETs within placental intervillous space. Placental NETs are elevated in HIV+ve compared to HIV-ve groups (Moodley et al. 2020b). Notably, neutrophil invasion into the placenta is expected as it is a highly vascularized zone and the site of inflammation. Moreover, similar homing has been noted in other inflammatory vascularized areas such as the kidney and lungs (Papayannopoulos 2018). This could imply an immuno-defensive role of NETs in the placenta where syncytiotrophoblasts may also be involved in HIV entrapment (Moodley et al. 2020a).

#### Pre-eclampsia does not affect the concentration of serum neutrophil extracellular traps

We report no significant difference in the level of circulating NETs between N and PE pregnant women. There is a paucity of information on circulating NET concentration in PE. However, the production of ROS, particularly nitric oxide (NO), has been recently studied in PE in vivo. Similar to the NET expression in this study, the concentration of serum NO in PE serum was similar to that of normotensive controls (Kashinakunti et al. 2021).

Pre-eclampsia is linked to an exaggerated immune response with resultant oxidative stress. However, the target site of the exacerbated inflammation is the placenta—where insufficient myometrial spiral artery remodeling leads to an imbalance in ROS (Zhang et al. 1998; Gupta et al. 2005). Placental-derived ROS is linked to elevated NETs observed in PE placenta (Gupta et al. 2005; Moodley et al. 2020b), which highlights the specific role of NET production in the placenta, decidua and myometrium (Moodley et al. 2020b; Butterworth et al. 1991; Marder et al. 2016). In the placenta, NETs trap syncytiotrophoblast microparticles, apoptotic fragments and HIV proteins that predispose patients to hyper-inflammation and endothelial damage (Moodley et al. 2020c, 2020b; Gupta et al. 2005).

#### Neutrophil extracellular traps may be an early marker of early-onset pre-eclampsia in women who are positive for human immunodeficiency virus infection.

In the current study, NETs were elevated in EOPE+ve compared to LOPE+ve groups. An elevated neutrophil-to-lymphocyte ratio (NLR) is present in EOPE, with increased neutrophil activation, elastase release and subsequent NETosis (Aneman et al. 2020). Despite the release of pro-inflammatory factors into maternal circulation in both subsets, EOPE is linked to elevated soluble fms-like tyrosine kinase 1 (sFlt-1) (Hoeller et al. 2017; Zeisler et al. 2016). In PE, elevated sFlt-1 inhibits angiogenesis and the neutrophil upregulation of Flt-1 by the neutralization of vascular endothelial growth factor (VEGF). Subsequently, reduced VEGF-dependent neutrophil transmigration occurs (Krysiak et al. 2005)—which explains the elevated circulatory serum NETs in EOPE. Circulatory sFlt-1 is inversely related to defective uterine spiral artery remodeling (Vogtmann et al. 2021) and is implicated in the defective placentation, systemic inflammation and vascular damage of EOPE (Gardiner and Vatish 2017).

#### Concentration of neutrophil extracellular traps in non-pregnant vs. pregnant subgroups

Pregnancy is regarded as an inflammatory process; therefore, the current study investigated an additional normotensive non-pregnant control group (Sargent et al. 2006). The current study reports an elevation of serum NETs in the NNP+ve compared to N+ve, PE+ve and LOPE+ve subgroups.

This indicates that pregnancy decreases serum NET expression irrespective of pregnancy type, strengthening our hypothesis that neutrophils are stimulated to transmigrate to the placenta where their NETs directly interact with placental factors (Gupta et al. 2005).

Additionally, neutrophil-induced T cells (niT) express a pro-angiogenic phenotype and release VEGF, influencing placental vessel growth: trophoblast invasion and spiral artery remodeling. Supporting this is the difference between placental-derived and maternal human leukocyte antigens (HLA’s) (Nelson et al. 1993). This shift in immunity promotes maternal immune tolerance by an induction of neutrophil regulatory GARP^+^CD127^lo^FOXP3^+^ phenotype of CD4+ T cells (which produce anti-inflammatory cytokines; IL-10 and IL-17) (Nadkarni et al. 2016). Early-onset PE is considered a more severe form of PE compared to LOPE (Raymond and Peterson 2011). In the current study, no significant difference in serum NETs between NNP and EOPE was found, which indicates that the hyper-stimulated NETs in the placenta could be undergoing reverse migration into the maternal circulation, enhancing the threat to maternal hyper-inflammation in EOPE (Buckley et al. 2006).

#### There is an inverse correlation between neutrophil extracellular traps and body mass index in late-onset pre-eclampsia without human immunodeficiency virus infection

There was a significant inverse correlation of maternal BMI with cit-H3 serum concentration in the LOPE-ve group (Fig. 8A). Since BMI is a risk factor for PE (Wang et al. 2013), this finding clearly indicates a link between BMI and serum NETs, particularly in LOPE-ve individuals. High glucose reduces ROS production on peripheral neutrophils, which subsequently explains the correlation of reduced NETs with increasing BMI (Perner et al. 2003). This study also showed that placental weight and baby birth weight are significantly positively correlated in the following groups: PE-ve, EOPE-ve and LOPE-ve (Fig. 8B–D): the EOPE-ve group had a strong positive correlation with r = 0.9999.

### Strengths

This study is novel in reporting JAM-C immunoexpression within the placental conducting and exchange villi based on pregnancy type, HIV status and HIV co morbid with PE and normotensive pregnancy. The strengths of the study are: (1) the in vivo analysis of NETs, (2) the novelty in investigating the relationship between serum NETs characterized by HIV status, pregnancy type and gestational age of PE, (3) using cit-H3 as the NET-specific marker and (4) including a secondary control group (non-pregnant).

### Limitations

This study was limited in the retrospective design and subsequent lack of information on antiretroviral therapy impact on JAM-C expression in HIV+ve patients. An ideal placental study would be one where multiple biopsies are taken from different sites of the placenta. Therefore, this study is limited to representing information pertaining to JAM-C immunoexpression in the central placental regions from the fetal side of the placenta with attempts to cover the full depth of the placenta. The current study was limited in the following area: sample size, especially regarding the non-pregnant group (and these results may be treated as preliminary data, which were not stratified by HIV status).

## Conclusion

Individually, PE and HIV had elevated JAM-C expression. However, PE comorbid HIV synergistically downregulated JAM-C expression. Resultantly, the enhanced neutrophil r-TM may be an effective marker of PE in HIV-infected women. The hypoxic microenvironment of PE does not significantly affect circulatory NET expression. However, in HIV+ve women, NET expression was exacerbated in EOPE compared to LOPE. Thus, NET may be a specific effective early marker of EOPE development in HIV-infected women.

## Future recommendations

The outcomes of this study potentiate further investigations of a larger cohort using fresh samples. Maternal blood should be analyzed across the gestational period for placenta-derived neutrophils with the specific neutrophil r-TM marker. These outcomes may potentiate the role of JAM-C and/or reverse neutrophil migration as an early diagnostic marker of PE in HIV+ve women. Further prospective investigations using fresh samples of a larger cohort are required to explore a multi-panel analysis on neutrophil count and function. The outcome of such a study could add to the body of knowledge in assisting the clinical diagnosis and management of PE.

## Data Availability

The authors declare that the data supporting the findings of this study are available within the paper and raw datasets are available from the corresponding author upon reasonable request.
